# Macrophages engulf apoptotic and primary necrotic thymocytes through similar phosphatidylserine‐dependent mechanisms

**DOI:** 10.1002/2211-5463.12584

**Published:** 2019-02-13

**Authors:** Zsófia Budai, László Ujlaky‐Nagy, Gréta Nikoletta Kis, Miklós Antal, Csaba Bankó, Zsolt Bacsó, Zsuzsa Szondy, Zsolt Sarang

**Affiliations:** ^1^ Department of Biochemistry and Molecular Biology Faculty of Medicine University of Debrecen Hungary; ^2^ Department of Biophysics and Cell Biology Faculty of Medicine University of Debrecen Hungary; ^3^ Department of Anatomy, Histology and Embryology Faculty of Medicine University of Debrecen Hungary; ^4^ Department of Biophysics and Cell Biology Faculty of Medicine and Faculty of Pharmacy University of Debrecen Hungary; ^5^ Department of Basic Medical Sciences Faculty of Dentistry University of Debrecen Hungary

**Keywords:** apoptosis, macrophages, phagocytosis, phosphatidylserine, primary necrosis

## Abstract

One of the major roles of professional phagocytes is the removal of dead cells in the body. We know less about the clearance of necrotic cells than apoptotic cell phagocytosis, despite the fact that both types of dead cells need to be cleared together and necrotic cells appear often in pathological settings. In the present study, we examined phagocytosis of heat‐ or H_2_O_2_‐killed necrotic and apoptotic thymocytes by mouse bone marrow‐derived macrophages (BMDMs) *in vitro* and found that the two cell types are engulfed at equal efficiency and compete with each other when added together to BMDMs. Phagocytosis of both apoptotic and necrotic thymocytes was decreased by (a) blocking phosphatidylserine on the surface of dying cells; (b) inhibition of Mer tyrosine kinase, Tim‐4, integrin β3 receptor signaling, or Ras‐related C3 botulinum toxin substrate 1 activity; or (c) using BMDMs deficient for transglutaminase 2. Stimulation of liver X, retinoid X, retinoic acid or glucocorticoid nuclear receptors in BMDMs enhanced not only apoptotic, but also necrotic cell uptake. Electron microscopic analysis of the engulfment process revealed that the morphology of phagosomes and the phagocytic cup formed during the uptake of dying thymocytes is similar for apoptotic and necrotic cells. Our data indicate that apoptotic and necrotic cells are cleared via the same mechanisms, and removal of necrotic cells *in vivo* can be facilitated by molecules known to enhance the uptake of apoptotic cells.

**Abbreviations**
9cRA: 9‐*cis* retinoic acid
ATRA: all‐*trans* retinoic acid
BMDM: bone marrow‐derived macrophage
CD: cluster of differentiation
CFDA‐SE: carboxyfluorescein diacetate succinimidyl ester
CMTMR: 5‐(and‐6)‐(((4‐chloromethyl)benzoyl)amino)tetramethylrhodamine
GR: glucocorticoid receptor
LXR: liver X receptor
MerTK: Mer tyrosine kinase
MFG‐E8: milk fat globule‐EGF factor 8 protein
PS: phosphatidylserine
Rac1: Ras‐related C3 botulinum toxin substrate 1
RAR: retinoic acid receptor
RGD: arginylglycylaspartic acid
RXR: retinoid X receptor
TAM: Tyro3, Axl, Mer
TG2: transglutaminase 2
Tim‐4: T‐cell immunoglobulin mucin receptor 4

Every day billions of damaged or senescent cells die in our body and are replaced with new cells 1. One of the physiological cell death types is apoptosis characterized by detachment and shrinkage of the cell, condensation and fragmentation of nuclear content 2, maintenance of membrane integrity and display of ‘eat me’ signals such as phosphatidylserine (PS) 3, or disappearance of so‐called ‘don't eat me’ signals, such as cluster of differentiation (CD) 47 on the apoptotic cell surface 4. Apoptosis can be activated by a wide range of stimuli, which trigger either the cell death receptor or the mitochondrial pathway of apoptosis 5, 6. Apoptosis is considered an immunologically silent process, since not only do apoptotic cells fail to induce inflammation, but uptake of apoptotic cells was shown to actively suppress the inflammatory program in engulfing macrophages 7, 8. In contrast to apoptosis, necrosis is characterized by swelling of the cell and early membrane rupture 9 leading to release of the intracellular content, which can damage the surrounding tissues and initiate local inflammation 10, 11, 12. Several conditions can result in necrosis, such as exposure of cells to high temperature in burns, physical damage, hypoxia, viral infection or in the case of programmed necroptosis, cell death receptor ligation 13. Necrotic cells were also shown to display PS on their outer membrane leaflet, which is used for their uptake 14, 15. Similar to apoptotic cells, engagement of PS receptors on the surface of macrophages elicits an anti‐inflammatory response, but this effect is overridden by the noxious cell content released during cell necrosis 14, 16, 17. Efficient clearance of necrotic cells in the organism helps to resolve the wounded area and the initiated inflammation. Apoptotic cells can also lose membrane integrity and undergo secondary necrosis in cases in which they are not cleared from the tissues properly 18. In this case, the accumulating secondary necrotic cells initiate local inflammation, which can lead to the development of autoimmune diseases in the long term 19, 20.

Macrophages are considered to be the primary phagocytes responsible for clearing dead cells in most organs. Macrophages are equipped with a battery of receptors to recognize, bind and engulf apoptotic cells. Among others, these receptors include the direct PS receptor T‐cell immunoglobulin mucin receptor 4 (Tim‐4), stabilin‐2, brain‐specific angiogenesis inhibitor 1, and receptors, such as Mer tyrosine kinase (MerTK), integrin α_v_β_3_ and its co‐receptor transglutaminase 2 (TG2), that bind to PS through various bridging molecules 21. Interestingly, the CD36–α_v_β_3_ integrin receptor complex was reported to recognize and bind externalized PS during necrotic cell uptake as well 16. The phagocytic receptors participating in the uptake of apoptotic cells activate two evolutionarily conserved signaling pathways, both of which trigger the activity of the small G protein Rac that regulates actin reorganization and lamellipodia formation during phagocytosis 22. Despite the shared integrin receptors considerable differences were found in the uptake of the two cell types. On one hand, it was suggested that necrotic cells compete with apoptotic ones much more efficiently than the other way around 14, though surprisingly apoptotic cells are engulfed more quickly than necrotic ones 17. On the other, in sharp contrast, it was indicated by another laboratory that macrophages have distinct modes for apoptotic and necrotic cell recognition, and they do not compete with each other upon simultaneous exposure 23. While uptake of apoptotic cells takes place via well‐defined portals in macrophages, where phagocytic receptors assemble together to mediate efficient uptake of a number of dead cells into tight‐fitting phagosomes 24, necrotic cell phagocytosis seems to take place via a macropinocytosis‐like process generating spacious macropinosomes and an abundant number of pseudopods 25. Activation of nuclear receptor liver X (LXR), retinoic acid (RAR), retinoid X (RXR) 26 and glucocorticoid (GR) receptors 26, 27, 28 in macrophages leads to upregulation of several of the above‐mentioned apopto‐phagocytic genes and results in enhanced apoptotic cell clearance.

While we consider apoptosis and necrosis as two different cell death forms, under pathological conditions they very often appear together and have to be cleared simultaneously 29, 30, 31, 32, 33, 34. Thus in this study, we decided to reinvestigate the mechanism and preference in the uptake of apoptotic and necrotic cells by studying the engulfment of serum‐starved apoptotic and heat‐ or H_2_O_2_‐killed necrotic thymocytes by bone marrow‐derived macrophages (BMDMs). Our data indicate that apoptotic and heat‐ or H_2_O_2_‐treated necrotic thymocytes are cleared by macrophages via similar PS‐dependent mechanisms and this clearance can be enhanced by nuclear receptor agonists known to induce apoptotic cell phagocytosis. In addition, the necrotic and apoptotic cells are engulfed at equal efficiency and compete with each other suggesting similar uptake mechanisms.

## Materials and methods

### Reagents

All reagents were obtained from Sigma‐Aldrich (Budapest, Hungary) except when indicated otherwise.

### Experimental animals

The experiments were carried out with 4‐week‐old or 2‐ to 4‐month‐old C57B6 mice. In some experiments, TG2^+/+^ and TG2^−/−^ mice were used. Mice were maintained in specific pathogen‐free conditions in the Central Animal Facility and all animal experiments were approved by the Animal Care and Use Committee of University of Debrecen (DEMÁB).

### BMDM cell culture and treatment

Bone marrow progenitors were obtained from the femurs of 2‐ to 4‐month‐old mice. Femurs were washed with sterile physiological saline. Cells were allowed to differentiate for 6 days in DMEM supplemented with 10% FBS, 10% conditioned medium derived from L929 cells as a source of macrophage colony‐stimulating factor, 2 mm glutamine, 100 U·mL^−1^ penicillin and 100 mg·mL^−1^ streptomycin at 37 °C in 5% CO_2_. Non‐adherent cells were washed away after 3 days. BMDMs were treated with the RAR agonist all‐*trans* retinoic acid (ATRA; 1 μm), the RXR agonist 9‐*cis* retinoic acid (9cRA; 1 μm), the LXR agonist GW3965 (Tocris, Bioscience, Bristol, UK; 1 μm) or the GR agonist dexamethasone acetate (1 μm) to activate nuclear receptors, or vehicle (0.5% DMSO) for 24 h. In the inhibitory experiments, 1 μm BMS777607 Tyro3, Axl, Mer (TAM) receptor tyrosine kinase inhibitor or 100 μm NSC23766 small GTP binding protein Ras‐related C3 botulinum toxin substrate 1 (Rac1) inhibitor (Tocris) was used for 24 h. For blocking integrins, either 2 mg·mL^−1^ arginylglycylaspartic acid (RGD) peptide or 4 μg·mL^−1^ fluorescein isothiocyanate hamster anti‐mouse CD61 antibody (BD Biosciences, Franklin Lakes, NJ, USA) was used for 1 h before addition of the apoptotic or necrotic cells. Four micrograms per milliliter Tim‐4 monoclonal antibody [54 (RMT4‐54)], phycoerythrin (Thermo Fisher Scientific, Waltham, MA, USA) was used for 1 h to block the PS receptor Tim‐4 on the surface of BMDMs.

### Apoptosis and necrosis induction and PS masking on thymocytes

**Figure 1:**
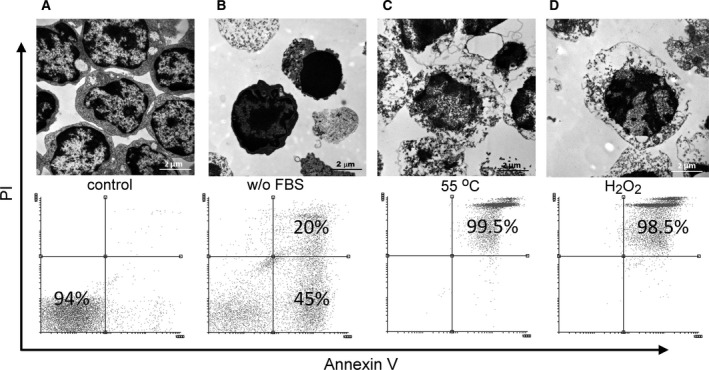
Morphology and viability of target cells. Transmission electron microscopic images and propidium iodide/annexin V staining of target cells. (A) Thymocytes immediately after isolation are considered as viable cells. (B) Apoptosis induced by serum deprivation for 24 h is characterized with condensed nuclei. According to the propidium iodide staining, both early and late apoptotic cells are included. (C,D) Incubation at 55 °C for 20 min (C) or with 1 mm H_2_O_2_ for 24 h (D) induced necrosis with early swelling and propidium iodide positivity. Scale bar: 2 μm.

Thymi were collected from 4‐week‐old C57B6 mice, and thymocytes were isolated and cultured for 24 h (10^7^ cells·mL^−1^) in DMEM supplemented with 2 mm glutamine, 100 U·mL^−1^ penicillin, and 100 mg·mL^−1^ streptomycin. To avoid potential effects of apoptosis inducers on the uptake of apoptotic cells, apoptosis of thymocytes was induced by growth factor withdrawal 35 by incubating the cells in serum‐free medium for 24 h, as we described previously 36. To generate necrotic target cells, thymocytes were incubated either at 55 °C for 20 min 23 or with 1 mm H_2_O_2_ for 24 h 37. Since isolated thymocytes show spontaneous cell death after 24 h of incubation 38, freshly isolated thymocytes were used as living cell controls in the experiments. Propidium iodide/annexin V labeling was used to determine the percentage of apoptotic and necrotic cells and transmission electron microscopy was used to confirm the cell death mechanism (Fig. 1A–D). Ten micromolar carboxyfluorescein diacetate succinimidyl ester (CFDA‐SE; Thermo Fisher Scientific) or 0.5 μm Cell Tracker deep red dye (Thermo Fisher Scientific) was used to label apoptotic and necrotic cells as indicated below. PS was blocked by Alexa Fluor® 647‐conjugated annexin V (Thermo Fisher Scientific) on the surface of apoptotic and necrotic thymocytes.

### *In vitro* phagocytosis assay

BMDMs were plated in 24‐well plates (3 × 10^5^/well) and co‐incubated with fluorescently stained apoptotic or necrotic cells (CFDA‐SE, deep red). Thymocytes were added to the BMDMs at the indicated (dead cells per macrophage) ratios. Since 100% of heat‐ or H_2_O_2_‐treated cells were necrotic, but in the apoptotic samples 30–40% cells were living, before the phagocytosis experiments we verified the viability stage of the apoptotic and necrotic cell population and normalized the target cell number to have the same amount of apoptotic and necrotic cells during phagocytosis assays. Phagocytosis was allowed to proceed for the indicated time periods at 37 °C. After coculture, thymocytes were washed away extensively and macrophages were detached by trypsinization. Percentage of macrophages engulfing dead cells was determined on a BD Biosciences FACSCalibur flow cytometer. In competition experiments differently stained apoptotic (CFDA‐SE) and necrotic (deep red) thymocytes were added together at different ratios (1 : 1, 1 : 3, 1 : 6, 1 : 9) to macrophages. For determining phagocytic preference, macrophages were exposed to stained (CFDA‐SE) apoptotic or necrotic thymocytes for 30 min, and then were further exposed to labeled apoptotic or necrotic cells. After coculture, thymocytes were washed away extensively and macrophages were detached by trypsinization. Percentage of macrophages engulfing dead cells was analyzed on a FACSCalibur flow cytometer.

### Laser scanning cytometry

BMDMs were plated into (IBIDI, Madison, WI, USA) eight‐well chamber slides (30 000 macrophages per well) and were stained with Hoechst 33342. Fluorescently labelled apoptotic (CFDA‐SE) and necrotic (deep red) thymocytes were added into the BMDMs immediately before the measurement. During the measurement, cells were kept in an IBIDI incubator at 37 °C with 5% CO_2_ air and 90% humidity. Images were made using an Olympus (Tokyo, Japan) IX‐71 inverted microscope and a video file was generated from these images using imagej software (NIH, Bethesda, MD, USA).

### Confocal microscopy

Prior to measurement BMDMs were plated into IBIDI eight‐well chamber slides (30 000 macrophages per well) and were stained with (5‐(and‐6)‐(((4‐chloromethyl)benzoyl)amino)tetramethylrhodamine (CMTMR); Thermo Fisher Scientific). Fluorescently labelled apoptotic (CFDA‐SE) and necrotic (deep red) thymocytes were added into the BMDMs immediately before the measurement. Time‐lapse movies were made using Zeiss (Oberkochen, Germany) LSM510 confocal laser scanning microscope. Images were taken every 10 s at 140 nm per pixel resolution. The region of interest was extracted and exported with 16 frames·s^−1^ speed, yielding a compressed video 160 times the actual speed of the process of phagocytosis.

### Transmission electron microscopy

For transmission electron microscopy apoptotic and necrotic thymocytes were collected and subsequently fixed with 1% glutaraldehyde in 0.1 m cacodylate buffer, and postfixed with 2% OsO_4_ in the same buffer. For analysis of phagocytosis, 5‐day‐old BMDMs on a glass slide were fed with apoptotic and necrotic thymocytes for 30 min and subsequently fixed with 1% glutaraldehyde in 0.1 m cacodylate buffer, and postfixed with 2% OsO_4_ in the same buffer. Slide samples were then dehydrated and stained with uranyl acetate and lead citrate and observed under a Zeiss EM900 electron microscope.

### Statistical analyses

All the data are representative of at least three independent experiments. Values are expressed as mean ± SD. Statistical analysis was performed using an unpaired Student's *t* test.

## Results and Discussion

### The uptake of both apoptotic and necrotic cells is PS‐, MerTK‐, Tim‐4‐, integrin β3‐ and TG2‐dependent

**Figure 2:**
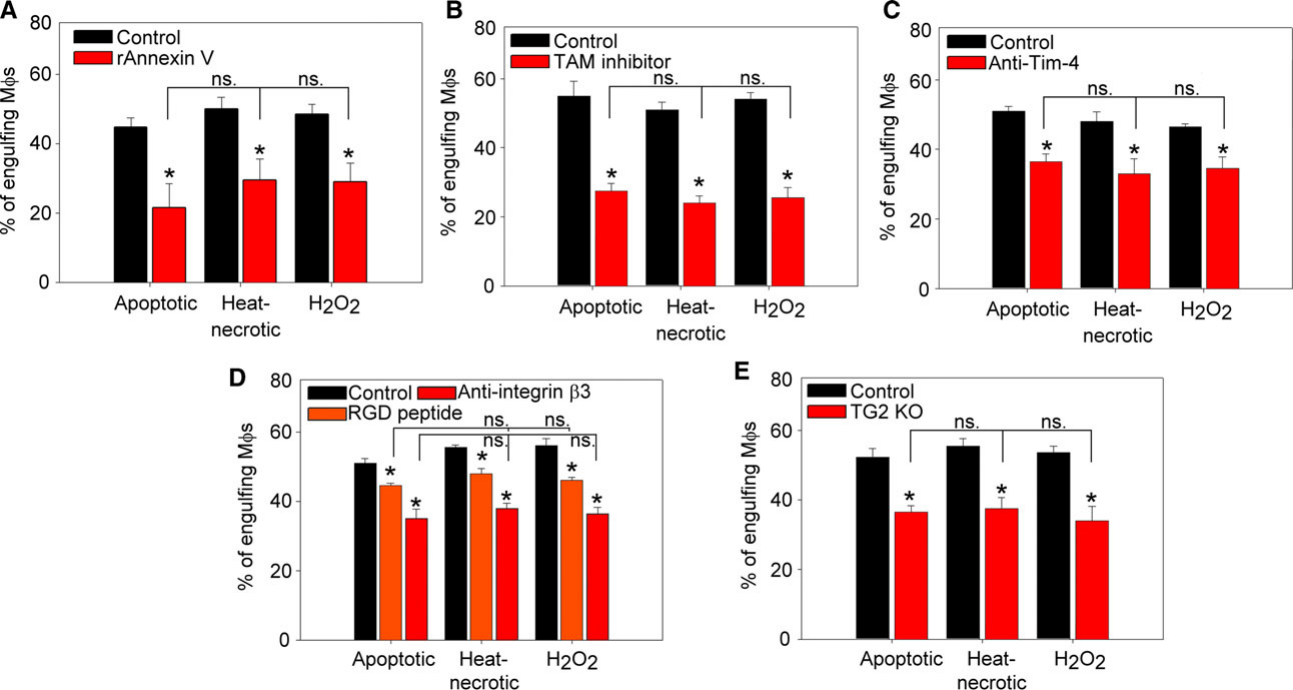
PS‐dependent mechanisms participate in the uptake of both apoptotic and primary necrotic cells. (A) Phagocytosis of control or recombinant annexin V‐exposed fluorescently labeled apoptotic or necrotic thymocytes by BMDMs added in 5 : 1 target cell : macrophage ratio. (B–E) Phagocytosis of fluorescently labelled apoptotic or necrotic thymocytes added to BMDMs in 5 : 1 target cell : macrophage ratio after treating macrophages with 1 μm BMS777607 TAM kinase inhibitor (B) for 24 h, or in the presence of 4 μg·mL^−1^ anti‐Tim‐4 antibody (C), 4 μg·mL^−1^ anti‐integrin β3 antibody or 2 mg·mL^−1^ RGD peptide (D). (E) Phagocytosis by macrophages derived from TG2 knockout mice was also tested. Apoptosis and necrosis were induced as described in Materials and methods. Phagocytosis was allowed for 60 min. Results are expressed as mean ± SD of three independent experiments (*significantly different from its own control, *P* < 0.05 determined by unpaired Student's *t* test). ns: not significant; MΦ?s: macrophages.

The exposure of PS on the surface of apoptotic cells is an important primary signal recognized by phagocytes. Our results using the high‐affinity PS‐binding protein annexin V labeling confirmed the results from others that PS appeared on the surface of both heat‐killed and H_2_O_2_‐exposed thymocytes (Fig. 1). Thus, we decided to test whether the uptake of apoptotic and necrotic thymocytes is dependent on PS via performing blocking experiments with annexin V. Masking of PS on the surface of necrotic and apoptotic thymocytes by recombinant annexin V treatment significantly decreased the uptake of both cell types (Fig. 2A) indicating a role of PS in the phagocytosis of necrotic and apoptotic cells. Previously, we detected the expression of several phagocytic receptors (integrin α_v_, β_1_, β_3_, β_5_, MerTK, Tim‐4, Stabilin‐2, CD14, and CD36) in mouse BMDMs 26. Among these, thrombospondin–CD36–α_v_β_3_ complex, CD14, and CD36 were already demonstrated to participate in the necrotic cell uptake as well 20.

In our experiments, we aimed to test the requirement for the other PS‐dependent receptors in the necrotic cell uptake. MerTK is a member of the TAM family of receptor tyrosine kinases and can bind the PS indirectly via bridging molecules (e.g. growth arrest specific 6, protein S) 39, 40. Inhibition of MerTK by using the BMS777607 TAM receptor tyrosine kinase inhibitor in BMDMs resulted in 50% reduction of both necrotic and apoptotic thymocyte uptake (Fig. 2B). We also tested the involvement of the direct PS receptor Tim‐4 in necrotic cell uptake by blocking the receptor with an anti‐Tim‐4 antibody and observed a similarly decreased necrotic and apoptotic thymocyte uptake (Fig. 2C). α_v_β_3_ integrin receptors also can recognize PS on the surface of apoptotic and necrotic cells in an indirect way with the help of bridging molecules [e.g. milk fat globule‐EGF factor 8 protein (MFG‐E8)] 41. Blockade of receptor α_v_β_3_ function by RGD peptides or by an anti‐mouse CD61 antibody resulted in an effective inhibition of the uptake of both apoptotic and necrotic thymocytes by BMDMs (Fig. 2D). Previously we have reported that TG2 can act as a β_3_ integrin co‐receptor in the context of MFG‐E8 binding; thus, it can contribute to the proper phagocytosis of apoptotic cells 24. Using BMDMs derived from TG2^−/−^ mice, we again observed a similar reduction in the uptake of apoptotic and necrotic cells (Fig. 2E). Altogether, our observations indicate that the same PS‐recognizing receptors participate in the uptake of apoptotic and primary necrotic cells.

### Triggering of various nuclear receptors enhances the phagocytosis of both apoptotic and necrotic cells

**Figure 3:**
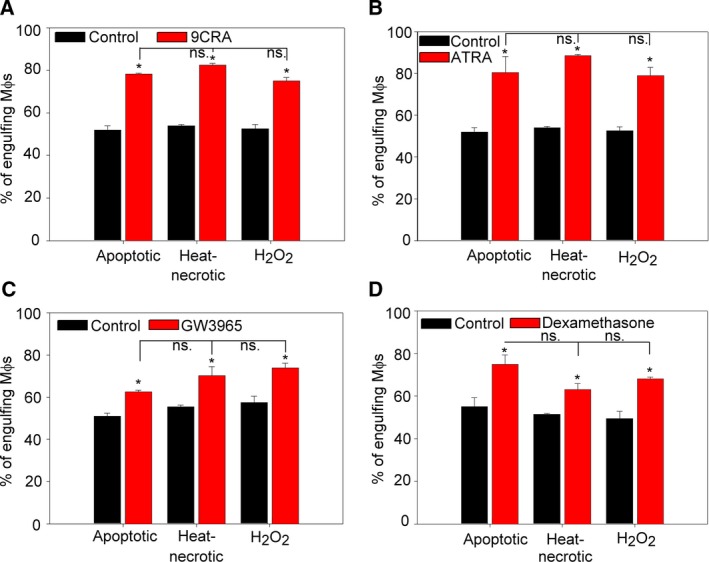
Stimulation of nuclear receptors increases the uptake of both apoptotic and necrotic thymocytes by BMDMs. BMDMs were treated with the RXR agonist 9cRA (A), with the RAR agonist ATRA (B), with the LXR agonist GW3965 (C) or with the GR agonist dexamethasone acetate (D) all in 1 μm concentrations for 24 h. Apoptotic or necrotic thymocytes were added to BMDMs in 5 : 1 target cell : macrophage ratio. Apoptosis and necrosis were induced as described in Materials and methods and phagocytosis was allowed for 60 min. Results are expressed as mean ± SD of three independent experiments (*significantly different from its own control, *P* < 0.05 determined by unpaired Student's *t* test). ns: not significant.

Previously we have demonstrated that during engulfment the lipid content of apoptotic cells triggers the LXR receptor of the macrophage which, in response, upregulates the expression of MerTK and retinaldehyde dehydrogenases leading to retinoid synthesis, which then contributes to the upregulation of further phagocytic receptors and finally results in enhanced apoptotic cell engulfment 26. The retinoid‐dependent phagocytic receptors included TG2, Tim‐4, and stabilin‐2 in BMDMs. In addition, it was also demonstrated that activation of the GR also leads to an enhanced apoptotic cell uptake mainly via MerTK‐dependent mechanisms 26, 27. That is why we decided to compare how triggering these nuclear receptors affects the uptake of apoptotic and necrotic thymocytes by BMDMs. For this purpose, macrophages were treated with 9cRA (RXR agonist; Fig. 3A), ATRA (RAR agonist; Fig. 3B), GW3965 (LXR agonist; Fig. 3C) or dexamethasone (GR agonist; Fig. 3D) for 24 h before exposing them to apoptotic or necrotic thymocytes. According to our results triggering of all these nuclear receptors in BMDMs enhances the uptake of both apoptotic and necrotic thymocytes suggesting that nuclear receptor ligation‐induced phagocytosis enhancement is not restricted only to apoptotic cell uptake.

### Apoptotic and necrotic thymocytes compete with each other during *in vitro* phagocytosis

**Figure 4:**
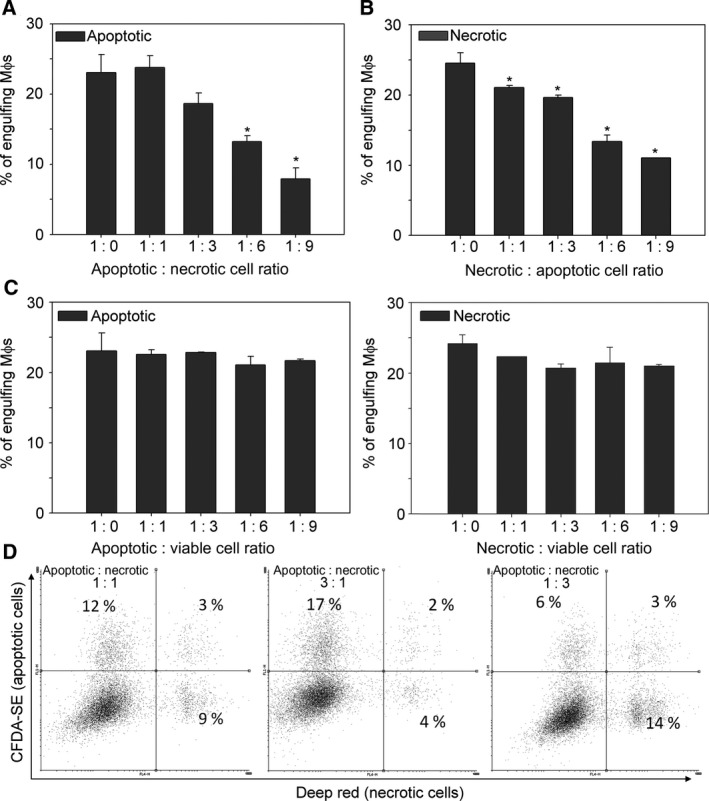
Apoptotic and necrotic thymocytes compete with each other for uptake by BMDMs. (A) Uptake of apoptotic cells in the presence of increasing amounts of necrotic cells. (B) Uptake of necrotic cells in the presence of increasing amounts of apoptotic cells. (C) Uptake of apoptotic and necrotic cells in the presence of increasing amounts of viable cells. BMDMs were exposed to differently stained apoptotic (CFDA‐SE), heat‐necrotic (deep red) and non‐stained viable thymocytes at the indicated ratios. (D) Representative flow cytometry scatter plots of BMDMs engulfing differently stained target cells at the indicated apoptotic : necrotic cell ratios. The initial number of apoptotic or necrotic cells was fixed to 1 million and only the amount of the other cell type was increased. Apoptosis and necrosis were induced as described in Materials and methods. Phagocytosis was allowed for 30 min to make increased phagocytosis detectable even in the presence of significantly enhanced target cell number. Results are expressed as mean ± SD of four independent experiments (*significantly different from its own control, *P* < 0.05 determined by unpaired Student's *t* test).

If the same receptors participate in the uptake of both apoptotic and necrotic cells, uptake of the two cell types should compete with each other. To investigate this possibility, we performed short‐term phagocytosis competition assays for 30 min in which mouse BMDMs were fed with differently stained apoptotic and/or necrotic thymocytes in various apoptotic–necrotic cell ratios. As shown in Fig. 4A,B, necrotic thymocytes were engulfed as efficiently as apoptotic ones, and they competed for the uptake equally well when added together to macrophages. In contrast, viable cells are not phagocytosed and they do not compete with dead cell phagocytosis (Fig. 4C). During the flow cytometry measurements, we observed, however, that only a small percentage of macrophages engulfed both apoptotic and necrotic cells when added together (Fig. 4D). Therefore, we went on to determine whether uptake of one cell type influences the further phagocytic preference of BMDMs.

### Macrophages use the same phagocytic portal for engulfing apoptotic and necrotic cells

**Figure 5:**
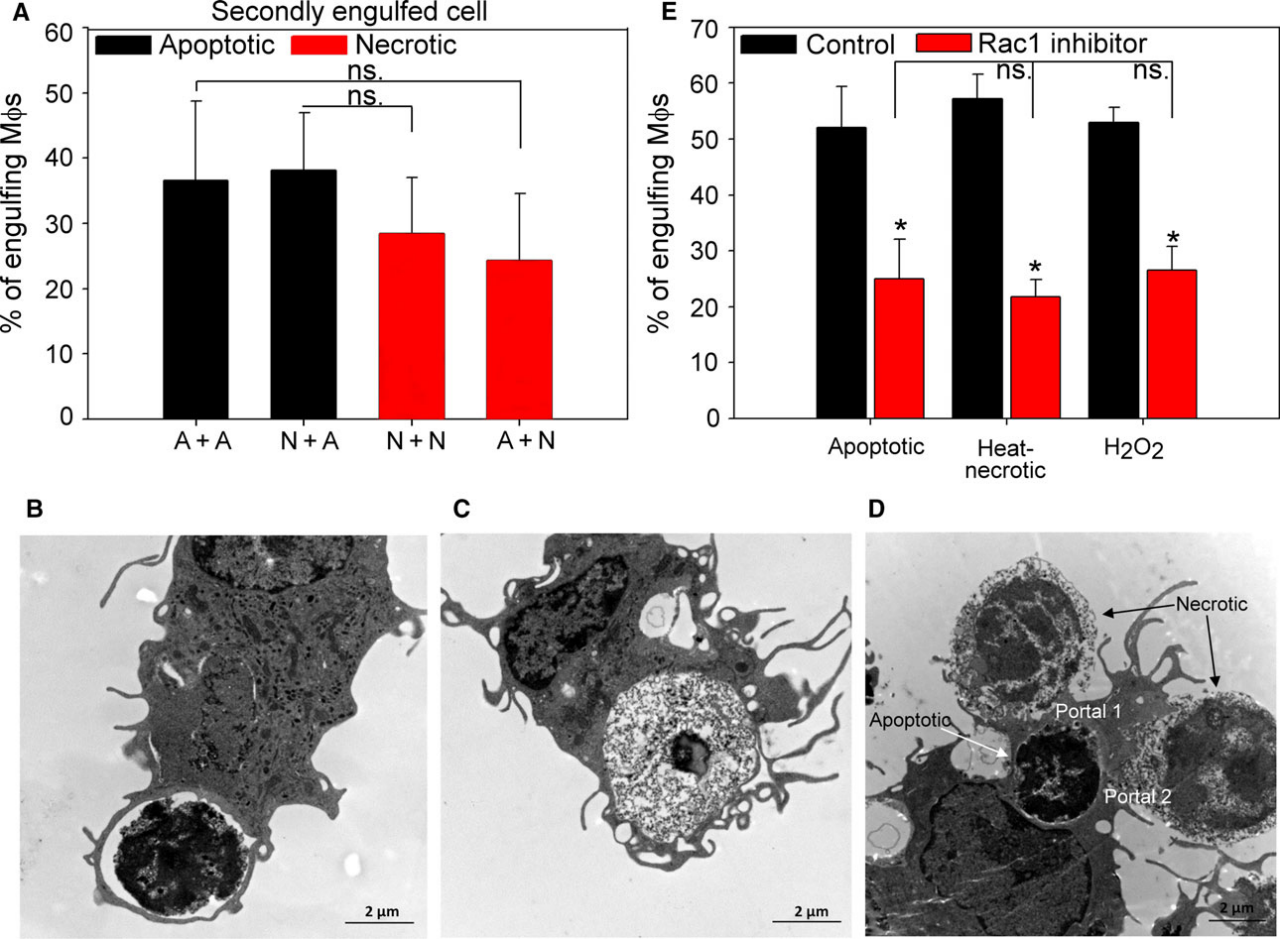
Apoptotic and necrotic thymocytes are engulfed via the same phagocytic portal and their uptake is Rac1‐dependent. (A) BMDMs were exposed first to either apoptotic or necrotic thymocytes for 30 min, and then these cells were washed away and phagocytosis was followed with the same amount, but differently stained apoptotic or necrotic cells for an additional 30 min. Apoptosis and necrosis were induced as described in Materials and methods. The graph shows the fraction of the BMDMs that engulfed the secondly added cells as the percentage of macrophages that phagocytosed the firstly added target cells. Results are expressed as mean ± SD of four independent experiments. (B–D) Representative transmission electron microscopic images of BMDMs engulfing apoptotic (B), heat‐killed necrotic (C) cells, or both types of dead cells simultaneously (D). (E) BMDMs were treated with the NSC23766 Rac1 inhibitor (100 μm) for 24 h and subsequently exposed to apoptotic or necrotic thymocytes in 5 : 1 target cell : macrophage ratio. Apoptosis and necrosis were induced as described in Materials and methods and phagocytosis was allowed for 60 min. Results are expressed as mean ± SD of four independent experiments (*significantly different from its own control, *P* < 0.05 determined by unpaired Student's *t* test). ns: not significant. Scale bar: 2 μm.

During engulfment the phagocytic receptors cluster laterally in the cell membrane forming phagocytic portals that can be used for the uptake of several apoptotic cells 24, 42, 43. Our observation could indicate that macrophages form a different phagocytic portal for the apoptotic and necrotic cells, and once the portal formation is induced, it will prefer the uptake of the related cell type. To investigate this possibility, macrophages were exposed first to fluorescently labeled either apoptotic or necrotic thymocytes for 30 min. Then they were washed and exposed to either apoptotic or necrotic cells stained differently for an additional 30 min. As shown in Fig. 5A, we found that within those macrophages that already engulfed a target cell, the percentage of the uptake of the second cell type was independent of the previously engulfed cells indicating that the preassembled phagocytic portals for the two target cell type may be similar or the same. Interestingly, the uptake of necrotic cells, when added following the prefeeding, was slightly lower as compared to apoptotic cell phagocytosis, but this lower uptake was independent of the first cell type taken up. Thus we concluded that the low simultaneous uptake of apoptotic and necrotic cells observed in Fig. 4D might be related to the fact that during the short phagocytic uptake period mostly only one target cell was taken up by macrophages.

In order to characterize the internalization mechanisms used by macrophages to engulf apoptotic and necrotic cells, we studied the morphological characteristics of BMDMs during phagocytosis by transmission electron microscopy. During the individual uptake of both apoptotic and necrotic thymocytes, the engulfing pseudopods similarly followed tightly the contour of the target particle (Fig. 5B–C and Fig. S1). Moreover, the two types of dead cells can be taken up via the same phagocytic portal (Fig. 5D, Videos [Link], [Link], and Fig. S1A–C) suggesting that similar preassembled receptor clusters might play a role in their phagocytosis. Previously, experiments using electron microscopic visualization of early apoptotic and necrotic L929 fibroblast cell phagocytosis demonstrated two different uptake mechanisms for the uptake of these cells 25. The apparent contradictions might be resolved by taking the size of the targets into consideration. L929 cells were shown to form small apoptotic bodies that were surrounded by pseudopods and internalized into tight‐fitting phagosomes while large necrotic cells are engulfed piece‐by‐piece via a macropinocytosis‐like process. In our case, the small size of thymocytes, as compared to L929 cells, might enable the utilization and formation of similar pseudopods and phagosomes during apoptotic and necrotic cell uptake.

A key participant of phagocytosis of apoptotic cells is the small GTPase Rac1, which regulates the redistribution of actin to the membrane ruffles and is activated by all the phagocytic signaling pathways 21. Inhibition of Rac1 by the NSC23766 small GTP binding protein Rac1 inhibitor inhibited the uptake of necrotic cells as well (Fig. 5E) indicating that not only the phagocytic receptors, but the signaling pathways activated by them must be the same in the uptake of apoptotic and necrotic cells. Altogether our data indicate that both heat‐ and H_2_O_2_‐treated necrotic thymocytes express PS and are taken up by PS‐dependent mechanisms, as was indicated previously 22. We identified several PS‐dependent phagocytic receptors, such as MerTK, integrins, TG2, and Tim‐4, which are known to participate in the uptake of apoptotic cells, as participating in necrotic cell uptake as well. Nuclear receptor stimulation, known to induce the expression of these phagocytic receptors 26, 27, 28 and consequently the uptake of apoptotic cells, also induced the engulfment of both types of necrotic cells further proving the involvement of the same phagocytic receptors. Prevention of the necrotic cell uptake by Rac1 inhibition indicated that not only the receptors, but also the signaling pathways of apoptotic and necrotic cell uptake are shared. Moreover, the electron microscopic images also showed that the same phagocytic portals are opened for the uptake of both apoptotic and necrotic cells. In accordance, our results also demonstrate that necrotic cells are engulfed as efficiently as apoptotic ones and they compete for uptake equally well when added together to macrophages. What is more, the uptake of the first cell type (apoptotic or necrotic) does not decide the preference for the second cell type taken up. These observations indicate that under *in vivo* pathological settings, when very often both apoptotic and necrotic cells can be detected in the affected tissue, both cell types will be cleared with similar efficiency. Effective clearance of dead cells plays a central role in the prevention of long‐term pathological events 44, 45. We have recently reviewed the therapeutic possibilities, such as that LXR, peroxisome proliferator‐activated receptor gamma, GR receptor ligation or macrolide antibiotic administration can enhance the uptake of apoptotic cells 46. Our present observations indicate that many of these approaches might be effective in stimulating the necrotic cell clearance as well. Dead cells can be removed not only by professional phagocytes, but by neighboring non‐professional phagocytes as well. Interestingly, these cells utilize the same mechanisms for efferocytosis that macrophages use 47. To support our findings, administration of MFG‐E8, a bridging molecule that links dead cell surface PS to the α_v_β_3_ integrin and its co‐receptor TG2 on the phagocytes 24, 41, accelerated the recovery after myocardial infarction 48, a condition where necrotic and apoptotic cells were shown to be engulfed by neighboring cardiac myofibroblast 32.

## Conclusions

Altogether our results indicate that primary necrotic cell phagocytosis seems morphologically similar to apoptotic cell uptake and the two processes involve TIM‐4, MerTK, integrin a_v_β_3_, TG2, and Rac1 molecules on the site of macrophages and PS on the dead cells surface. GR, RAR or LXR receptor activation augments both necrotic and apoptotic cell clearance, and therefore clinical therapies targeting apoptotic cell clearance might also enhance necrotic cell uptake, which can contribute to the success of these strategies.

## Conflict of interests

The authors declare no conflict of interest.

## Author contributions

ZB performed the majority of experiments, analyzed the data, and participated in the writing of the manuscript; L‐NU, MA, ZB, CB, and GNK participated in the microscopic experiments; ZS and ZS designed and coordinated the research, and participated in the writing of the manuscript. All authors read and approved the final manuscript.

## Supporting information


**Fig. S1.** (A–C) Representative transmission electron microscopic images of BMDMs engulfing apoptotic and heat‐killed necrotic thymocytes at the same site. (D–E) Representative transmission electron microscopic images showing that BMDMs form tight‐fitting phagosomes around both the engulfed apoptotic and heat‐killed necrotic thymocytes. Scale bar: 5 μm (A, B) or 2 μm (C–E).

**Video S1.** Fluorescence live‐cell imaging of apoptotic and necrotic cell engulfing BMDMs by laser scanning microscopy. Apoptotic and necrotic thymocytes were added to BMDMs in 5 : 1 target cell : macrophage ratio. Apoptosis and necrosis were induced as described in Materials and methods. Apoptotic thymocytes are labeled with green, necrotic thymocytes with red and the nuclei of BMDMs with blue colors. Arrows point to macrophages that took up an apoptotic and a necrotic cell at the same site.

**Video S2.** Fluorescence live‐cell imaging of apoptotic and necrotic cell engulfing BMDMs by confocal microscopy. Apoptotic and necrotic thymocytes were added to BMDMs in 5 : 1 target cell : macrophage ratio. Apoptosis and necrosis were induced as described in Materials and methods. Apoptotic thymocytes are labeled with green, necrotic thymocytes with blue and BMDMs with red colors. In the middle there is a macrophage that took up firstly an apoptotic then a necrotic cell at the same site. Note that apoptotic and necrotic cells interact at several sites with macrophages but uptake happens only at one site.
