# Supplementary figures and images for: Macrophages engulf apoptotic and primary necrotic thymocytes through similar phosphatidylserine‐dependent mechanisms
Source: FEBS Open Bio. 2019 Feb 13;9(3):446–56. doi: 10.1002/2211-5463.12584 (PMC6396166; doi:10.1002/2211-5463.12584)

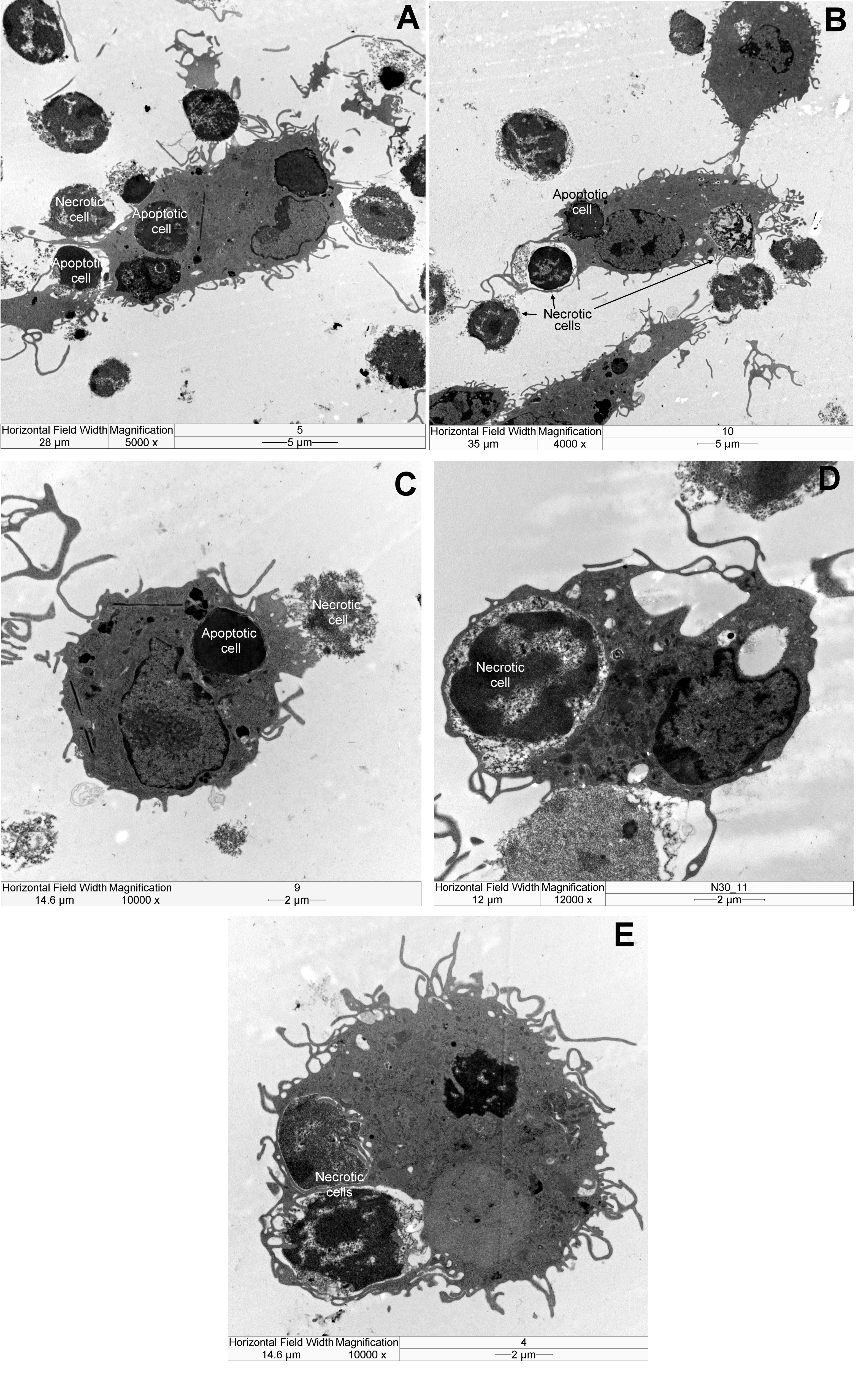

Supplement: Supplementary file 1 — Fig. S1. (A–C) Representative transmission electron microscopic images of BMDMs engulfing apoptotic and heat‐killed necrotic thymocytes at the same site. (D–E) Representative transmission electron microscopic images showing that BMDMs form tight‐fitting phagosomes around both the engulfed apoptotic and heat‐killed necrotic thymocytes. Scale bar: 5 μm (A, B) or 2 μm (C–E). [file FEB4-9-446-s001.tif]
