# Supplementary material for: Macrophages engulf apoptotic and primary necrotic thymocytes through similar phosphatidylserine‐dependent mechanisms
Source: FEBS Open Bio. 2019 Feb 13;9(3):446–56. doi: 10.1002/2211-5463.12584 (PMC6396166; doi:10.1002/2211-5463.12584)
Supplement: Supplementary file 4 [file FEB4-9-446-s004.doc]

**Supplementary Fig 1.** (A-C) Representative transmission electron microscopic images of BMDMs engulfing apoptotic and heat-killed necrotic thymocytes at the same site. (D-E) Representative transmission electron microscopic images showing that BMDMs form tight-fitting phagosomes around both the engulfed apoptotic and heat-killed necrotic thymocytes.

**Supplementary video 1.** Fluorescent live-cell imaging of apoptotic and necrotic cell engulfing BMDMs by laser-scanning microscopy. Apoptotic and necrotic thymocytes were added to BMDMs in 5:1 target cell:macrophage ratio. Apoptosis and necrosis were induced as described in Materials and Methods. Apoptotic thymocytes are labeled with green, necrotic thymocytes with red and the nuclei of BMDMs with blue colors. Arrows point to macrophages that took up an apoptotic and a necrotic cell at the same site.

**Supplementary video 2.** Fluorescent live-cell imaging of apoptotic and necrotic cell engulfing BMDMs by confocal microscopy. Apoptotic and necrotic thymocytes were added to BMDMs in 5:1 target cell:macrophage ratio. Apoptosis and necrosis were induced as described in Materials and Methods. Apoptotic thymocytes are labeled with green, necrotic thymocytes with blue and BMDMs with red colors. In the middle there is a macrophage which took up firstly an apoptotic than a necrotic cell at the same site. Note that apoptotic and necrotic cells interact at several sites with macrophages but uptake happens only at one site.
